# Decoding empagliflozin’s molecular mechanism of action in heart failure with preserved ejection fraction using artificial intelligence

**DOI:** 10.1038/s41598-021-91546-z

**Published:** 2021-06-08

**Authors:** Antoni Bayes-Genis, Oriol Iborra-Egea, Giosafat Spitaleri, Mar Domingo, Elena Revuelta-López, Pau Codina, Germán Cediel, Evelyn Santiago-Vacas, Adriana Cserkóová, Domingo Pascual-Figal, Julio Núñez, Josep Lupón

**Affiliations:** 1 Heart Institute, Hospital Universitari Germans Trias I Pujol, Carretera de Canyet S/N, 08916 Badalona, Spain; 2 Department of Medicine, Universitat Autònoma de Barcelona, Barcelona, Spain; 3 Centro de Investigación Biomédica en Red Enfermedades Cardiovasculares, (CIBERCV), Madrid, Spain; 4 Cardiology Department, Hospital Virgen de la Arrixaca, IMIB-Arrixaca and University of Murcia, Murcia, Spain; 5 Centro Nacional de Investigaciones Cardiovasculares (CNIC), Madrid, Spain; 6 Cardiology Department, Hospital Clínico Universitario de Valencia, INCLIVA, Departamento de Medicina, Universitat de València, Valencia, Spain

**Keywords:** Computational biology and bioinformatics, Systems biology, Cardiology, Medical research

## Abstract

The use of sodium-glucose co-transporter 2 inhibitors to treat heart failure with preserved ejection fraction (HFpEF) is under investigation in ongoing clinical trials, but the exact mechanism of action is unclear. Here we aimed to use artificial intelligence (AI) to characterize the mechanism of action of empagliflozin in HFpEF at the molecular level. We retrieved information regarding HFpEF pathophysiological motifs and differentially expressed genes/proteins, together with empagliflozin target information and bioflags, from specialized publicly available databases. Artificial neural networks and deep learning AI were used to model the molecular effects of empagliflozin in HFpEF. The model predicted that empagliflozin could reverse 59% of the protein alterations found in HFpEF. The effects of empagliflozin in HFpEF appeared to be predominantly mediated by inhibition of NHE1 (Na^+^/H^+^ exchanger 1), with SGLT2 playing a less prominent role. The elucidated molecular mechanism of action had an accuracy of 94%. Empagliflozin’s pharmacological action mainly affected cardiomyocyte oxidative stress modulation, and greatly influenced cardiomyocyte stiffness, myocardial extracellular matrix remodelling, heart concentric hypertrophy, and systemic inflammation. Validation of these in silico data was performed in vivo in patients with HFpEF by measuring the declining plasma concentrations of NOS2, the NLPR3 inflammasome, and TGF-β1 during 12 months of empagliflozin treatment. Using AI modelling, we identified that the main effect of empagliflozin in HFpEF treatment is exerted via NHE1 and is focused on cardiomyocyte oxidative stress modulation. These results support the potential use of empagliflozin in HFpEF.

## Introduction

Heart failure with preserved ejection fraction (HFpEF) currently comprises almost half of all heart failure (HF) cases, and no evidence-based treatment has been proven to improve outcomes. Current therapy for HF with reduced ejection fraction (HFrEF) is based on modulation of the neurohormonal activation, i.e. inhibiting deleterious activation of the renin–angiotensin–aldosterone and sympathetic nervous systems and amplifying the protective natriuretic peptide system. However, such strategies have not been so successful in several landmark trials in HFpEF^1–3^.

Sodium-glucose co-transporter 2 inhibitors (SGLT2i) are a new class of drugs under evaluation for use in HF. They have yielded better than anticipated clinical benefit in HFrEF, with and without diabetes, in two expedited landmark clinical trials—namely, the DAPA-HF with dapagliflozin^4^ and Emperor-Reduced with empagliflozin^5^. At this time, pivotal clinical trials are underway with both dapagliflozin and empagliflozin in HFpEF.

The mechanism of action of SGLT2i includes blockade of the SGLT2 receptor at the proximal renal tubule. SGLT2 receptors are responsible for 90% of glucose reabsorption into the bloodstream, and their inhibition leads to glycosuria and reduced glycaemic levels^6,7^. However, SGLT2i-induced glycaemic control alone seems insufficient to explain the reported cardiovascular benefits, and a comprehensive mechanistic explanation of the extra-renal cardioprotective effects of SGLT2i remains elusive. Recent evidence suggests that the benefit of SGLT2i in HFrEF may also be mediated by the sodium-hydrogen exchanger (NHE)^8,9^.

Artificial intelligence (AI) is an interdisciplinary field in which computational and mathematical models are used to disentangle key interactions within complex biological networks^10^ and is particularly well-suited for investigating the mechanisms underlying drug effects. Our group has previously used AI to enhance our understanding of the mechanisms of action of sacubitril/valsartan^11^ and empagliflozin in HFrEF^9^. In the present study, we used AI to unravel the effects of empagliflozin in HFpEF at the molecular level.

## Results

### Intensity of empagliflozin’s therapeutic effect in HFpEF

**Figure 1:**
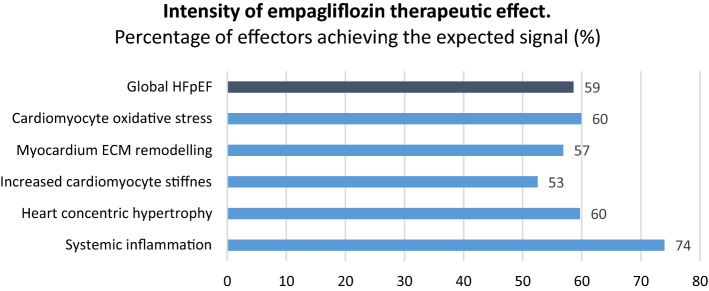
Intensity of empagliflozin therapeutic activity in HFpEF. Data are reported as percentage of effectors modulated by empagliflozin in the mathematical models, in HFpEF (as a whole) and in each individual pathophysiological motif measured. This value indicates empagliflozin’s capability to reverse the protein alterations occurring in HFpEF.

Figure 1 shows the intensity of the predicted effect of empagliflozin, measured as the percentage of protein effectors modulated by empagliflozin in HFpEF as a whole, and in each pathophysiological motif. The AI models predicted that empagliflozin would significantly modulate (> 50%) each pathophysiological motif in HFpEF. Furthermore, evaluation of the impact on HFpEF as a whole, indicated that empagliflozin treatment would reverse up to 59% of the identified protein alterations. These results suggest potential benefits of empagliflozin in HFpEF treatment.

### Mechanism of action elucidation

ANN-based prediction revealed that the activity of empagliflozin in HFpEF would primarily occur through inhibition of NHE1 (Na^+^/H^+^ exchanger 1), with less prominent roles played by the other targets (SGLT2 and NHE3) (Table 1). Notably, NHE1 is the only target protein of empagliflozin that is expressed in cardiomyocytes and vascular cells.

**Table 1:** Efficacy of empagliflozin on individual targets in HFpEF.

Empagliflozin target	HFpEF	Systemic inflammation	Oxidative stress	Heart concentric hypertrophy	Myocardial ECM remodelling	Cardiomyocyte stiffness
SGLT2	30%	28%	32%	29%	30%	28%
NHE1	77%	4%	82%	4%	3%	14%
NHE3	4%	4%	55%	5%	4%	5%

Columns show the artificial neural networks (ANN) score obtained (in %) for each target in HFpEF (as a whole) and in each individual pathophysiological motif.

HFpEF, heart failure with preserved ejection fraction; SGLT2, sodium-glucose co-transporter 2; NHE, Na^+^/H^+^ exchanger; ECM, extracellular matrix.

**Figure 2:**
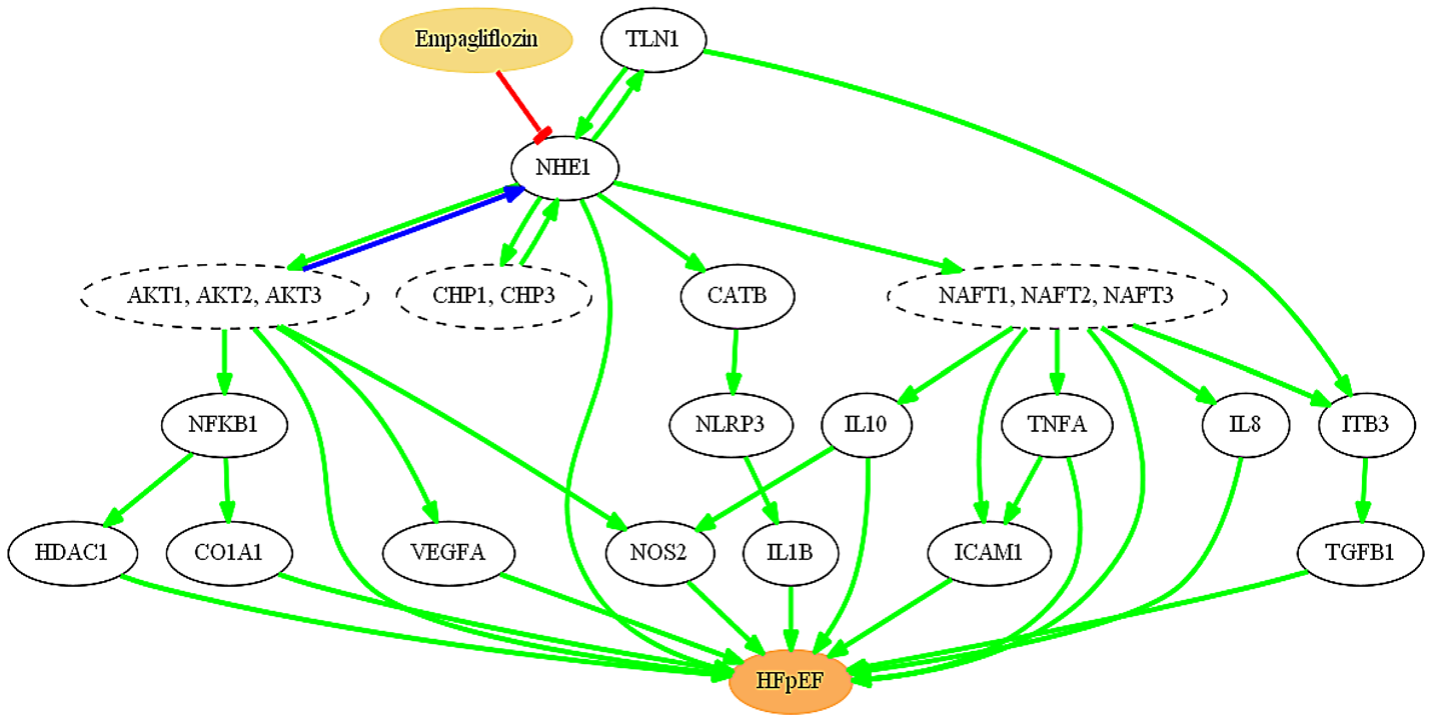
Graphical representation of the identified mechanism of action of empagliflozin in HFpEF. These paths were predicted by mathematical modelling, and biologically contextualized. Green lines indicate activations; red lines indicate inhibition; and blue lines indicate either activation or inhibition (cell-dependent effect). Broken lines show nodes that contain more than one protein, all of which participate in the mechanism of action in the same way.

Analysis of the AI mathematical models elucidated a cascade of the molecular interactions and key proteins most likely involved in the therapeutic effects of empagliflozin in HFpEF (Fig. 2). A set of 250 biologically plausible solutions were calculated, and the most probable mechanism of action was identified with a mean accuracy of 94%. Several major findings can be derived from Fig. 2. First, it appears that one distinctive result of empagliflozin is the modulation of oxidative stress mechanisms (an upstream event in HFpEF development), via the cardiomyocyte-expressed target NHE1 through various effectors, including AKTs (RAC-alpha/beta/gamma serine/threonine-protein kinases), nuclear factors of activated T-cells signalling (NFATs), and nitric oxide synthase (NOS2) (Fig. 2).

Second, it is clear that systemic inflammation is highly affected by empagliflozin’s mechanism of action. The use of this drug in our simulation altered several proteins implicated in inflammation, including tumour necrosis factor-α (TNF-α), interlukin-1 β (IL-1β), intercellular adhesion molecule-1 (ICAM1), and the NACHT, LRR, and PYD domains-containing protein 3 (NLRP3) inflammasome, leading to reduced inflammation.

Lastly, downstream of empagliflozin-mediated NHE1 inhibition, we observed strong modulation of cardiomyocyte stiffness, myocardial extracellular matrix remodelling, and heart concentric hypertrophy mechanisms. These empagliflozin-inhibited myocardial alterations are mediated by vascular endothelial growth factor A (VEGFA), NFκB-induced collagen alpha-1 chain (CO1A1), transforming growth factor β1 (TGF-β1), NFATs, and interleukin-10 (IL-10). In the models, these events were identified as downstream consequences of the more direct effects of NHE1 inhibition on oxidative stress mechanisms (Fig. 2).

### Clinical validation of empagliflozin’s predicted effect

To validate the selected protein effectors identified in silico, we measured their circulating concentrations in a prospective cohort of patients with HFpEF, before and 12 months after empagliflozin treatment (mean dose, 10 mg/day). The patients’ characteristics are displayed in Supplementary material online, Table S6. Briefly, 80% were male, the mean age was 69 ± 12 years, and mean LVEF was 58 ± 6%.

We evaluated the kinetics of NOS2, NLPR3, and TGF-β1 during the 12 months of empagliflozin treatment. The mean delta changes observed were: − 14% for NOS2 (baseline: 290 ± 106 pg/mL; 12 months: 249 ± 102 pg/mL), − 6% for NLPR3 (baseline: 299 ± 70 pg/mL; 12 months: 281 ± 67 pg/mL), and − 17% for TGF-β1 (baseline: 313 ± 27 pg/mL; 12 months: 259 ± 17 pg/mL). These findings were in line with the AI-generated data.

### Empagliflozin efficacy benchmark versus other drugs for HFpEF

ANN analyses revealed that HFpEF modulation by empagliflozin is similar to the effectiveness shown by ACEI, ARNI, β-blockers, and MRAs, and superior to ARBs (Table 2). Systemic inflammation in HFpEF was predominantly modulated by ACEI and ARNI; extracellular matrix remodelling by MRA, ACEI, and ARNI; and stiffness-related processes by β-blockers and ARNI. Thus, the main differential effect of empagliflozin compared to other treatments for HFpEF is its potential to directly modulate oxidative stress.

**Table 2:** Efficacy benchmarking of empagliflozin and other therapeutic treatments in HFpEF.

Treatment	HFpEF	Systemic inflammation	Oxidative stress	Heart concentric hypertrophy	Myocardial ECM remodelling	Cardiomyocyte stiffness
Empagliflozin	72%	9%	65%	20%	17%	7%
ACEI	71%	70%	33%	3%	80%	4%
ARB	28%	38%	34%	11%	68%	32%
ARNI	77%	51%	36%	56%	79%	57%
BB	65%	4%	16%	4%	21%	73%
MRA	71%	4%	3%	19%	81%	16%

The columns show the artificial neural networks (ANN) score obtained (in %) for each drug in HFpEF (as a whole) and in each individual pathophysiological motif.

HFpEF, heart failure with preserved ejection fraction; ACEI, angiotensin-converter enzyme inhibitors; ARB, angiotensin receptor blockers; ARNI, angiotensin-receptor neprilysin inhibitors; BB, β-Blockers; MRA, mineralocorticoid receptor antagonists; ECM, extracellular matrix.

## Discussion

HFpEF is characterized by normal systolic function and altered diastolic function. Diastolic dysfunction is mainly caused by defects in myocardium relaxation, ventricular filling, and distensibility, all of which are associated with myocardial extracellular matrix remodelling, cardiac stiffness, and concentric cardiomyocyte hypertrophy^12^. In 2013, Paulus and Tschope^13^ postulated a novel paradigm for HFpEF, proposing that the impaired myocardial relaxation is triggered by systemic inflammation and elevated oxidative stress, which has recently been experimentally validated by others.

**Figure 3:**
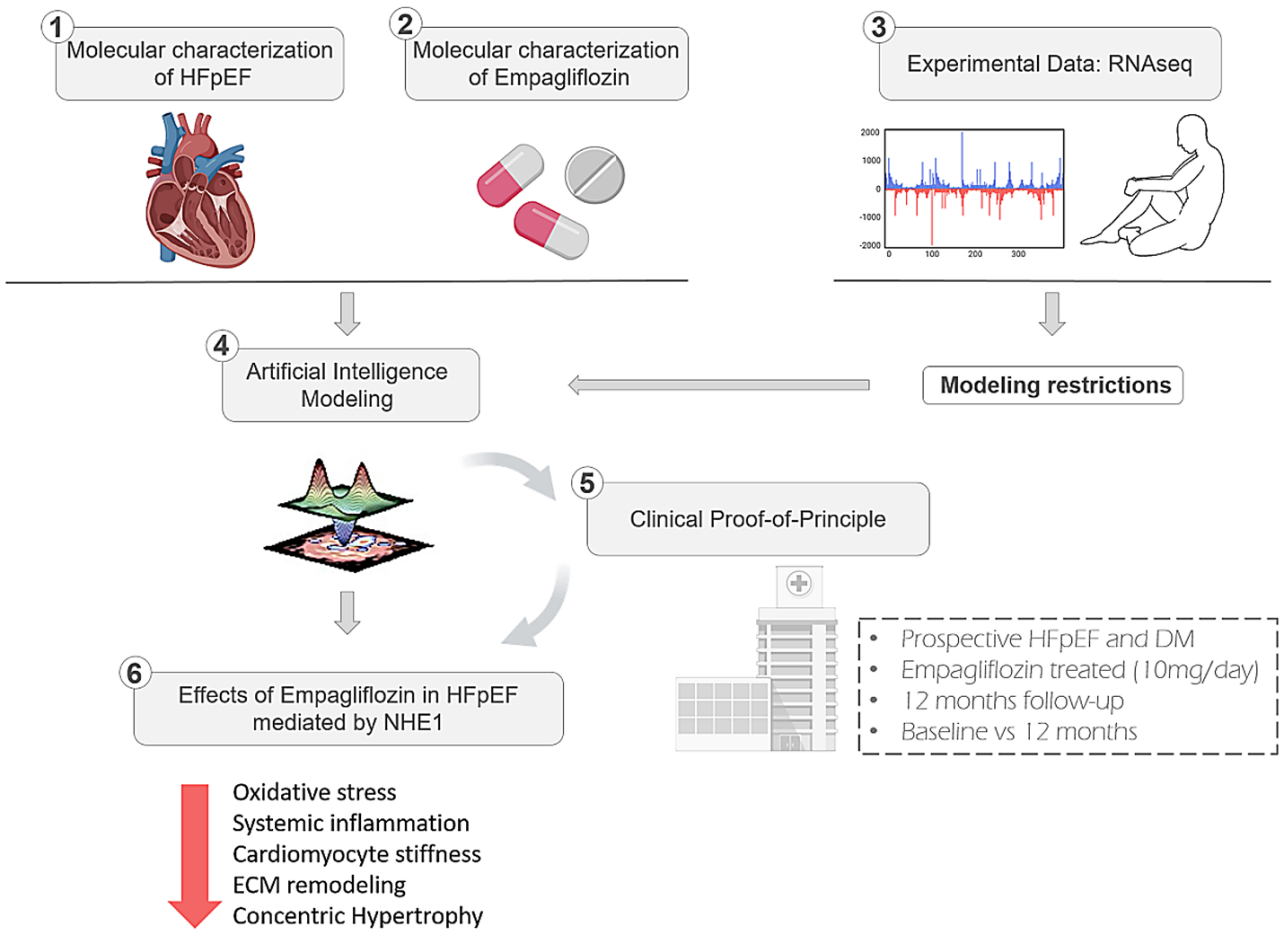
Graphical representation of the experimental designed followed in this study. First, we characterized the molecular profile of HFpEF and Empagliflozin (Panels 1 and 2). Next, we used experimental, RNAseq data to frame the behaviour of the future mathematical models (Panel 3). Then, we built a series of algorithms based on artificial intelligence techniques to elucidate the most prominent mechanism of action at play that could describe the clinical improvements observed in patients (Panel 4). Finally, we validated these findings in a small cohort of patients before and after being treated with empagliflozin, to delineate a specific signalling cascade (Panels 5 and 6). ECM: Extracellular matrix; HFpEF: heart failure with preserved ejection fraction; DM: diabetes mellitus.

In the present study, we examined empagliflozin’s mechanism of action using state-of-the-art AI modelling, considering all of the pathways involved in HFpEF progression (Fig. 3). Our analyses yielded several major findings. First, NHE1 is the main target of empagliflozin, mediating its benefits in HFpEF through direct effects on cardiomyocyte oxidative stress and cytoplasm acidification. Second, empagliflozin inhibits several inflammatory factors, thus reducing the systemic inflammation contributing to the HFpEF phenotype. Third, the empagliflozin-mediated inactivation of NHE1 is also reflected in myocardial extracellular matrix remodelling, cardiomyocyte stiffness, and concentric hypertrophy. Lastly, other treatments used in HFpEF are less effective against oxidative stress, such that reduction of oxidative stress is the differential effect of empagliflozin. Collectively, these data support the value of empagliflozin for HFpEF treatment at the molecular level. The clinical relevance of these findings is presently under examination in the Emperor-Preserved Trial^14^.

### Empagliflozin modulates oxidative stress-related mechanisms in HFpEF

Empagliflozin has profound effects on the modulation of oxidative stress-related mechanisms in HFpEF, and NHE1 inhibition is the most prominent mediator of these effects through several mechanisms (Fig. 2). First, NHE1 inhibition impairs activation of the calcineurin B homologous protein (CHP1, CHP3) loop^15^, which is required for proper NHE1 activity^16^. This leads to decreased Ca^2+^-induced mitochondrial swelling and a reduced release of reactive oxygen species (ROS) in isolated cardiomyocytes^17^.

Second, inhibited NHE1 cannot initiate the signalling cascade that leads to activation of the AKT1/AKT2/AKT3 module^18^. Blockage of AKT prevents stimulation of inducible NOS2 production, thus reducing oxidative stress^18,19^. In turn, AKT-mediated NHE1 activation is cell-dependent, with AKT activating NHE1 in fibroblasts but inhibiting NHE1 in the myocardium^20^, thereby further modulating this ion exchanger.

Third, functional blockage of NHE1 also prevents activation of the NFAT1/NFAT2/NFAT3 pathway, which would result in the induction of NOS2 expression and nitric oxide production through activation of interleukin-10 (IL-10)^21,22^. Finally, NHE1 inhibition also interrupts the activation of HDAC1 (histone deacetylase 1) by NFκB (nuclear factor κ B) downstream of AKT, thus further reducing the oxidative stress produced by HDAC1-mediated acetylation defects^23,24^.

### Empagliflozin ameliorates myocardial extracellular matrix remodelling, cardiomyocyte stiffness, and concentric hypertrophy

NHE1 inactivation not only affects oxidative stress, but its effects are also reflected in myocardial extracellular matrix remodelling, cardiomyocyte stiffness and heart concentric hypertrophy through multiple mechanisms (Fig. 2). Impaired AKT activation^25^ leads to lower production of VEGFA (a growth factor implicated in cardiomyocyte stiffness and myocardial matrix remodeling^26–28^) and reduced NFκB-induced expression of CO1A1 (which is important in myocardial extracellular matrix remodelling in HFpEF^29–31^).

On the other hand, NHE1 acts in a loop with talin-1 (TLN-1)^20^, which is known to be involved in cytoskeletal connections. This loop makes TLN-1 unable to trigger integrin β-3 (IT-β3) induction, which regulates cellular senescence^32^, and inhibits further production of TGF-β1, which is essential for myocardial extracellular matrix development^33,34^.

Blockade of NFAT1 activation also leads to reduced IT-β3-TGF-β1 signalling, and downregulation of the pro-fibrotic and pro-stiffness cytokine IL-10^22,33^. Moreover, lack of activation of both NFAT2 and NFAT3 could have beneficial effects in HFpEF since both proteins are reportedly involved in cardiac concentric hypertrophy^35^.

### Empagliflozin modulates systemic inflammation in HFpEF

Through the inhibition of several inflammatory factors (Fig. 2), empagliflozin would substantially reduce the systemic inflammation contributing to the HFpEF phenotype. Inhibited NHE1 cannot activate CATB (cathepsin B), thus preventing induction of the NLRP3 inflammasome and the activation of IL-1β, which are directly involved in HFpEF^33^. TNF-α and ICAM1 are also involved in the inflammatory process, and also cannot be activated since their activators (NFAT2 and NFAT3) will no longer be activated by NHE1^36,37^.

In our present analysis, we also examined the molecular effects of other therapies that have been studied for use in HFpEF, but that have not shown incontrovertible clinical benefit. Empagliflozin displayed a more intense anti-oxidative stress effect than the other studied drugs, which were more impactful in the other studied motifs. Prospective clinical validation is required to determine whether empagliflozin’s effects could be further boosted by its combination with additional treatments, which is beyond the aims of the present study.

## Limitations

This study has several limitations. First, the presently applied AI modelling systems only utilize information that has already been described or uploaded in public repositories and demonstrated experimentally. As knowledge on SGLT2 is rapidly evolving, it is likely that the mechanism of action is even more multifactorial than currently envisioned. Second, the pharmacological effect of empagliflozin upon HFpEF (or more specifically oxidative stress) could encompass, and be affected by, other molecular signalling cascades, as well as by a wide variety of physiological affected pathways. Although our models show that oxidative stress signalling is the most robust pathway affected, it is likely that other pathways may also be involved. Finally, the clinical validation of selected bioflags was conducted in a small prospective cohort of ambulatory mildly symptomatic (all NYHA class I or II) patients with HFpEF, all of whom had a background of diabetes, which is currently the only indication for clinical use of empagliflozin. There remains a need for further investigation of the kinetics of such protein effectors under empagliflozin treatment in larger cohorts of patients with HFpEF, who have more severe symptoms (and likely more inflammation and oxidation), and with and without diabetes. The recent SOLOIST-WHF Trial showed benefit of sotagliflozin (an SGLT1 and SGLT2 inhibitor) in patients with diabetes and worsening HF (both HFrEF and HFpEF)^38^. Collectively, our present data provide appropriate proof-of-principle validation of the identified in silico data at the clinical level.

## Conclusions

Here we used AI modelling to examine the effects of empagliflozin in HFpEF. We found that the main effect of empagliflozin’s pharmacological action is exerted through inhibition of NHE1, and is focused on cardiomyocyte oxidative stress modulation. These effects could improve mitochondrial homeostasis and reduce ROS production in cardiomyocytes, thus potentially reducing downstream damaging mechanisms related to cardiac hypertrophy, stiffness, and remodelling. We also found that empagliflozin modulates systemic inflammation. The identified mechanism of action of empagliflozin is cogent within the context of current scientific knowledge and could prove to be an important asset in understanding the clinical benefit of SGLT2i in HFpEF patients.

## Materials and methods

### HFpEF molecular characterization

We first performed a thorough review of the current scientific evidence regarding specific proteins/genes relevant to HFpEF development, using PubMed (only considering articles in English). A summary of the pathways or motifs involved in HFpEF, and the number of effector proteins found in each motif, is presented in Supplementary material online, Table S1.

To better model HFpEF pathophysiology, we also searched for gene expression data in the Gene Expression Omnibus (GEO)^39,40^ and ArrayExpress^41^ public repositories, considering only studies performed in humans (*Organism: Homo sapiens*) using expression or protein arrays (*Series type: Expression profiling by array* and *protein profiling by protein array*)^42^. Transcriptomics data were analysed using the following tests: the Lilliefors (4 < n < 20) method to test the normality of samples; Student’s *t*-test or Wilcoxon rank-sum test to determine whether genes were differentially expressed between cohorts when samples showed a normal or non-normal distribution, respectively; and a multi-test correction approach with the false discovery rate (FDR), corrected using the Benjamini–Hochberg method. Genes displaying differential expression with an FDR < 0.05 in the dataset were deemed statistically significant. The genes/proteins that were differentially expressed in patients with HFpEF compared to healthy controls were incorporated into the mathematical models, and are presented in Supplementary material online, Table S2.

We also retrieved the molecular target profile of drugs of interest in HFpEF treatment. These drugs were evaluated at the target level, according to the DrugBank database^43^, to generate an efficacy benchmark for the use of empagliflozin in HFpEF treatment. The studied drugs included ACEI (enalapril, captopril, and ramipril), ARBs (losartan and valsartan), ARNI (sacubitril/valsartan), beta-blockers (bisoprolol), and MRA (spironolactone and eplerenone) (Supplementary material online, Table S3).

### Empagliflozin molecular characterization

In order to characterize empaglifozin and incorporate this information into its mechanism of action, an in-depth review of the following official documents:

- EMA—European Medicines Agency: European Public Assessment Report (EPAR).
- FDA—Food and Drug Administration: Multidisciplinary review and Chemistry review.
- Product Monograph.

Next, when available, target information was retrieved from specialised databases, including DrugBank (http://www.drugbank.ca/)^43^, Stitch (http://stitch.embl.de/)^44^, and SuperTarget (http://insilico.charite.de/supertarget/)^45^. Currently available publications regarding known targets and bioflags of empaglifozin were identified in PubMed (https://www.ncbi.nlm.nih.gov/pubmed/) on February, 2020. The specific search performed was the following:

- ("Empagliflozin" [Title]) AND ("heart failure" [Title/Abstract] AND "preserved".
- [Title/Abstract] AND "ejection" [Title/Abstract]) OR "HFpEF" [Title/Abstract]).

The articles obtained through this search were evaluated at the title and abstract level, and if molecular information was found, the articles were thoroughly reviewed to identify protein/gene candidates to be drug target candidates or bioflags. If the evidence of the modulation of a drug target candidate or bioflag by the treatments under study was judged not consistent enough to be assigned as a drug target/bioflag an additional PubMed search was performed specifically for the candidate, including all the protein names according to UniProtKB, If novel candidates were identified in this phase, they were included as drug targets/bioflags following the same criteria and protocol.

After this process of constructing the empagliflozin profile database, we input all of the targets/bioflags into the mathematical models and, in a completely unbiased manner, the algorithms find the most likely molecular pathways from these inputs to the clinical output desired. In this work, our algorithms found that SGLT2, NHE1 and NHE3 were the only inputs that could build a protein network that ultimately explain the clinical improvements observed in patients. And among them, only those models with NHE1 as the main target had a consistent and considerable predictive power.

The information regarding empagliflozin targets—including sodium/glucose co-transporter 2 (SGLT2) and other proposed targets, such as sodium/hydrogen exchangers 1 and 3 (NHE1 and NHE3)^9,46^—is shown in Supplementary material online, Table S4. The empagliflozin bioflags identified for this project are presented in Supplementary material online, Table S5.

### Artificial intelligence modelling

#### Artificial neural networks (ANNs)

ANNs are supervised algorithms that identify relationships between proteins (e.g. drug targets) and clinical elements of a network^47^, by inferring the probability that a specific relationship exists between two or more protein sets. The system attempts to find the shortest distance between two evaluated protein sets, thus generating a list of proteins ranked by their association with the defined pathophysiology. In this study, ANNs were used for the following analyses: (1) comparison of individual targets of empagliflozin in HFpEF (as a whole) and in each pathophysiological motif; (2) identification of empagliflozin bioflags in HFpEF; and (3) efficacy benchmarking of empagliflozin and other drugs that have been studied in HFpEF (ACEI, ARBs, beta-blockers, MRA, ARNI), including analyses of each drug’s relationship with the disease and the efficacy of empagliflozin compared to the other tested drugs.

#### Mathematical models

Finally, we developed mathematical models that integrated all available biological and pharmacological knowledge to simulate the behaviour of HFpEF and the predicted effects of empagliflozin, in terms of changes in protein activity within the human protein network. Briefly, the following steps were taken. First, input models were generated, which included information about empagliflozin and HfpEF and the protein/gene relationships identified in public databases. These models served to evaluate the inhibition or activation of one or more nodes within the protein network, and to determine how the signalling cascade was affected. Second, output models were generated, which included experimental RNAseq data (upregulated or downregulated genes/proteins) to frame the mathematical models with specific and direct information describing the disease (Supplementary material online, Fig. S1)^9,11^.

State-of-the-art AI technologies were used for modelling the complex protein network interaction structure and network activation signal flow. These included graph theory and statistical pattern recognition technologies, genetic algorithms, ANNs, dimensionality reduction techniques, and stochastic methods like Simulated Annealing and Monte Carlo^9,11^.

Advanced AI was used to perform two different types of analyses: (1) predicting the therapeutic intensity of empagliflozin in HfpEF (details provided in Supplementary material online), and (2) generating a detailed description of the molecular pathways involved in empagliflozin’s mechanism of action. The graphical representation was monitored to ensure representation of the proteins most modulated by empagliflozin in HfpEF. This approximation ensured in-depth visualization of the downstream effects of the drug over the different processes involved in HfpEF (Supplementary material online, Fig. S2).

#### Intensity of response

The “intensity” of the response is defined to measure the effect of a mechanism of action and compare it with others. The intensity is defined in this project as the amount of protein effectors or motives (#Eff) achieving the expected signal to ameliorate a disease. Assuming ${y}_{i}$ as the value achieved by a protein effector “i”, while ${v}_{i}$ is its expected effector sign (active or inactive) and $n$ is the total number of effectors described for a phenotype (HFpEF), we defined the number of effectors achieving the expected signal (*#Eff*). A drug (empagliflozin) is expected to revert the conditions of a disease phenotype. Consequently, a drug should inactivate the active protein effectors of a patho-phenotype and activate the inactive. Thus, the number of effectors of the pathology achieving an output signal with the opposite sign as that defined in the condition characterization is measured. Using Dirac’s d (i.e. d(0) = 1, and zero otherwise):

$$\# Eff = \mathop \sum \limits_{i = 1}^{n} \delta \left( {\frac{{v_{i} }}{{\left| {v_{i} } \right|}} + \frac{{y_{i} }}{{\left| {y_{i} } \right|}}} \right)$$

### Clinical validation of empagliflozin’s predicted effect

Ambulatory patients with diagnosis of HFpEF (according to ESC guideline criteria) and diabetes mellitus were prospectively included from November 2018 to May 2019 and followed for 12 months. At baseline, empagliflozin (fixed dose of 10 mg/day) was introduced in the treatment regimen. A blood sample was obtained at baseline (before empagliflozin initiation) and after 12 months with empagliflozin.

During the baseline visit, patients provided written consent for biomarker analyses and for the use of their clinical data for research purposes. The study was performed in compliance with the law protecting personal data in accordance with the international guidelines on clinical investigations from the World Medical Association’s Declaration of Helsinki. Informed consent was obtained from all participants. The ethics committee for research with medicinal products of the Germans Trias I Pujol University Hospital (CEIm; IRB00002131) approved the study (Code: GLUCOSUR-IC; ref. PI-18-163).

#### Bioflag assays

Blood samples were collected, and serum was obtained by centrifugation for 10 min at 1500 rcf and stored at − 80 °C. All samples were obtained between 09:00 am and noon using the same protocol at the first visit and at 12 months. All samples were processed in their first freeze–thaw cycle.

Commercially available ELISA kits were used, following the instructions of the manufacturer, for each validated protein in all patients. Data expressed as mean ± SEM.

##### Nitric oxide synthase 2, inducible (NOS2)

NOS2 was evaluated with the Human Nitric Oxide Synthase 2, Inducible ELISA kit (Abbexa Ltd, Cambridge, UK; code No. abx585063; lot No. E2007807K). The range of the assay is between 15.6 and 1000 pg/mL. Sensitivity is < 5.5 pg/mL.

##### NACHT, LRP and PYD domains containing protein 3 (NLRP3)

NLRP3 was measured by the Human NACHT, LRP and PYD domains containing protein 3 ELISA kit (Abbexa Ltd, Cambridge, UK; code No. abx516996; lot No. E2007562H). The assay range is 156–10,000 pg/mL and sensitivity is < 0.08 ng/mL.

##### Transforming Growth Factor Beta (TGFB1)

TGF-β1 was analysed by the Human Transforming Growth Factor Beta ELISA kit (Abbexa Ltd, Cambridge, UK; code No. abx153266; Lot No. E2007192B). The range of the assay is between 15.6 and 1000 pg/mL.

## Supplementary Information

Supplementary Information.

## Data Availability

The datasets generated during and/or analysed during the current study are available from the corresponding author on reasonable request.
