# Supplementary material for: Decoding empagliflozin’s molecular mechanism of action in heart failure with preserved ejection fraction using artificial intelligence
Source: Sci Rep. 2021 Jun 8;11:12025. doi: 10.1038/s41598-021-91546-z (PMC8187349; doi:10.1038/s41598-021-91546-z)

Decoding empagliflozin's molecular mechanism of action in heart failure with preserved ejection fraction using artificial intelligence

Antoni Bayes-Genis, MD PhD[1,2*]; Oriol Iborra-Egea, PhD[1,2*]; Giosafat Spitaleri, MD[1]; Mar Domingo, MD PhD[1,2]; Elena Revuelta-López, PhD[1,2]; Pau Codina, MD[1,2], Germán Cediel, MD PhD[1,2]; Evelyn Santiago-Vacas, MD PhD[1,2]; Adriana Cserkóová[1], Domingo Pascual-Figal, MD PhD[2,3,4], Julio Núñez, MD PhD[2,5]; Josep Lupón, MD PhD[1,2].

From: [1]Heart Institute, Hospital Universitari Germans Trias i Pujol, Badalona, Spain, and Department of Medicine, Universitat Autònoma de Barcelona, Barcelona, Spain; [2]Centro de Investigación Biomédica en Red Enfermedades Cardiovasculares, (CIBERCV), Madrid, Spain; [3]Cardiology Department, Hospital Virgen de la Arrixaca, IMIB-Arrixaca and University of Murcia, Murcia, Spain; [4]Centro Nacional de Investigaciones Cardiovasculares (CNIC), Madrid, Spain; 5 cardiology Department. Hospital Clínico Universitario de Valencia, INCLIVA. Departamento de Medicina. Universitat de València. Valencia, Spain.

Short Title: Molecular mechanism of action of empagliflozin in HFpEF

Word Count: 4725

* *These authors contributed equally*

Corresponding Author

Antoni Bayes-Genis, MD, PhD, FESC.

Head, Heart Institute. Hospital Universitari Germans Trias i Pujol.

Carretera de Canyet s/n 08916. Badalona (Barcelona), Spain.

E-mail: abayesgenis@gmail.com.

Supplementary Material

Supplementary Tables

Supplementary Table S1: Summary of the motifs identified as involved in HFpEF

HFpEF Motifs	# of effector proteins
**Myocardial extracellular matrix remodelling**	48
**Increased cardiomyocyte stiffness**	66
**Heart Concentric Hypertrophy**	21
**Systemic inflammation**	22
**Oxidative stress**	23
**Total of non-duplicated effector proteins**	**148**

Supplementary Table S2: Genes differentially expressed in patients with HFpEF. The level of expression of each gene is also indicated. Red arrow (↓) indicates downregulation and green arrow (↑) upregulation.

Uniprot code	Protein names	Gene names (primary)	Expression
Q9UL45	Biogenesis of lysosome-related organelles complex 1 subunit 6	BLOC1S6	↓
P61960	Ubiquitin-fold modifier 1	UFM1	↓
P10398	Serine/threonine-protein kinase A-Raf	ARAF	↑
Q8N3I7	Bardet-Biedl syndrome 5 protein	BBS5	↑
Q9NQ11	Polyamine-transporting ATPase 13A2	ATP13A2	↑
P19388	DNA-directed RNA polymerases I, II, and III subunit RPABC1	POLR2E	↑
O43765	Small glutamine-rich tetratricopeptide repeat-containing protein alpha	SGTA	↑
P09958	Furin	FURIN	↑
Q9Y6I3	Epsin-1	EPN1	↑
Q9Y3P4	Rhomboid domain-containing protein 3	RHBDD3	↑
Q9H159	Cadherin-19	CDH19	↓
O60243	Heparan-sulfate 6-O-sulfotransferase 1	HS6ST1	↓
Q969U6	F-box/WD repeat-containing protein 5	FBXW5	↑
P13489	Ribonuclease inhibitor	RNH1	↑
Q9NRA0	Sphingosine kinase 2	SPHK2	↓
P09486	SPARC	SPARC	↑
P98160	Basement membrane-specific heparan sulfate proteoglycan core protein	HSPG2	↓
Q9BU89	Deoxyhypusine hydroxylase	DOHH	↓
Q9UGT4	Sushi domain-containing protein 2	SUSD2	↓
Q6ZW13	Uncharacterized protein C16orf86	C16orf86	↓
Q14160	Protein scribble homolog	SCRIB	↓
P52943	Cysteine-rich protein 2	CRIP2	↓
Q5JSZ5	Protein PRRC2B	PRRC2B	↓
Q8NCU8	Mitoregulin	MTLN	↓
Q9BQD7	Adenine nucleotide translocase lysine N-methyltransferase	ANTKMT	↓
Q6ZW49	PAX-interacting protein 1	PAXIP1	↓
Q18PE1	Protein Dok-7	DOK7	↓
P07339	Cathepsin D	CTSD	↓
P24298	Alanine aminotransferase 1	GPT	↓
P41222	Prostaglandin-H2 D-isomerase	PTGDS	↓
Q9HCM7	Fibrosin-1-like protein	FBRSL1	↓
P49755	Transmembrane emp24 domain-containing protein 10	TMED10	↑

Uniprot code	Protein names	Gene names (primary)	Expression
Q3MIX3	Uncharacterized aarF domain-containing protein kinase 5	ADCK5	↓
P55273	Cyclin-dependent kinase 4 inhibitor D	CDKN2D	↓
Q9UQ03	Coronin-2B	CORO2B	↓
Q16643	Drebrin	DBN1	↓
Q96EL1	PAK4-inhibitor INKA1	INKA1	↓
Q6P1R4	tRNA-dihydrouridine(16/17) synthase [NAD(P)(+)]-like	DUS1L	↓
O15120	1-acyl-sn-glycerol-3-phosphate acyltransferase beta	AGPAT2	↓
Q9NW07	Zinc finger protein 358	ZNF358	↓
Q15334	Lethal(2) giant larvae protein homolog 1	LLGL1	↓
Q9BY27	Protein DGCR6L	DGCR6L	↓
O00411	DNA-directed RNA polymerase, mitochondrial	POLRMT	↓
Q96CT7	Coiled-coil domain-containing protein 124	CCDC124	↓
Q13574	Diacylglycerol kinase zeta	DGKZ	↓
Q99687	Homeobox protein Meis3	MEIS3	↓
Q9ULL5	Proline-rich protein 12	PRR12	↓
Q96RT7	Gamma-tubulin complex component 6	TUBGCP6	↓
O60645	Exocyst complex component 3	EXOC3	↓
O15047	Histone-lysine N-methyltransferase SETD1A	SETD1A	↓
Q92686	Neurogranin (Ng)	NRGN	↓
P15289	Arylsulfatase A	ARSA	↓
Q96KQ7	Histone-lysine N-methyltransferase EHMT2	EHMT2	↓
Q8IY17	Patatin-like phospholipase domain-containing protein 6	PNPLA6	↓
Q9H0X9	Oxysterol-binding protein-related protein 5	OSBPL5	↓
Q969V3	Nicalin	NCLN	↓
Q16584	Mitogen-activated protein kinase kinase kinase 11	MAP3K11	↓
O75147	Obscurin-like protein 1	OBSL1	↓
Q9H3S7	Tyrosine-protein phosphatase non-receptor type 23	PTPN23	↓
A6NKD9	Coiled-coil domain-containing protein 85C	CCDC85C	↓
Q92692	Nectin-2	NECTIN2	↓
Q7Z6L1	Tectonin beta-propeller repeat-containing protein 1	TECPR1	↓
Q9BRK4	Leucine zipper putative tumor suppressor 2	LZTS2	↓
Q14332	Frizzled-2	FZD2	↓
O14874	[3-methyl-2-oxobutanoate dehydrogenase [lipoamide]] kinase, mitochondrial	BCKDK	↓
Q9UIL1	Short coiled-coil protein	SCOC	↑
Q9UID3	Vacuolar protein sorting-associated protein 51 homolog	VPS51	↓
P47755	F-actin-capping protein subunit alpha-2	CAPZA2	↑
Q9C0C2	182 kDa tankyrase-1-binding protein	TNKS1BP1	↓
Q9BQJ4	Transmembrane protein 47	TMEM47	↑
Q96F63	Coiled-coil domain-containing protein 97	CCDC97	↓
Q5JPI3	Uncharacterized protein C3orf38	C3orf38	↑
Q6ZMK1	Cysteine and histidine-rich protein 1	CYHR1	↓
Q7Z7F7	39S ribosomal protein L55, mitochondrial	MRPL55	↓

Uniprot code	Protein names	Gene names (primary)	Expression
Q13330	Metastasis-associated protein MTA1	MTA1	↓
Q8N2F6	Armadillo repeat-containing protein 10	ARMC10	↑
P61009	Signal peptidase complex subunit 3	SPCS3	↑
Q9HAH7	Probable fibrosin-1	FBRS	↓
Q71SY5	Mediator of RNA polymerase II transcription subunit 25	MED25	↓
O43324	Eukaryotic translation elongation factor 1 epsilon-1	EEF1E1	↑
Q96BV0	Zinc finger protein 775	ZNF775	↓
Q9NZ01	Very-long-chain enoyl-CoA reductase	TECR	↓
O14908	PDZ domain-containing protein GIPC1	GIPC1	↓
Q9UJY5	ADP-ribosylation factor-binding protein GGA1	GGA1	↓
Q9Y6G5	COMM domain-containing protein 10	COMMD10	↑
Q7Z4F1	Low-density lipoprotein receptor-related protein 10	LRP10	↓
Q9BVC4	Target of rapamycin complex subunit LST8	MLST8	↓
Q15363	Transmembrane emp24 domain-containing protein 2	TMED2	↑
P61077	Ubiquitin-conjugating enzyme E2 D3	UBE2D3	↑
P36404	ADP-ribosylation factor-like protein 2	ARL2	↓
Q9BSJ2	Gamma-tubulin complex component 2	TUBGCP2	↓
A6NDV4	Transmembrane protein 8B	TMEM8B	↓
Q9HA77	Probable cysteine--tRNA ligase, mitochondrial	CARS2	↓
Q9NS73	MAP3K12-binding inhibitory protein 1	MBIP	↑
P51668	Ubiquitin-conjugating enzyme E2 D1	UBE2D1	↑
P54760	Ephrin type-B receptor 4	EPHB4	↓
P62834	Ras-related protein Rap-1A	RAP1A	↑
Q13459	Unconventional myosin-IXb	MYO9B	↓
Q04725	Transducin-like enhancer protein 2	TLE2	↓
Q6PCT2	F-box/LRR-repeat protein 19	FBXL19	↓
O00193	Small acidic protein	SMAP	↑
Q6DN90	IQ motif and SEC7 domain-containing protein 1	IQSEC1	↓
Q16740	ATP-dependent Clp protease proteolytic subunit, mitochondrial	CLPP	↓
Q04206	Transcription factor p65	RELA	↓
Q9UHX1	Poly(U)-binding-splicing factor PUF60	PUF60	↓
O60826	Coiled-coil domain-containing protein 22	CCDC22	↓
Q9BRQ8	Ferroptosis suppressor protein 1	AIFM2	↓
Q15819	Ubiquitin-conjugating enzyme E2 variant 2	UBE2V2	↑
Q96DE5	Anaphase-promoting complex subunit 16	ANAPC16	↑
Q03426	Mevalonate kinase	MVK	↓
P49458	Signal recognition particle 9 kDa protein	SRP9	↑
Q9BSL1	Ubiquitin-associated domain-containing protein 1	UBAC1	↓
O15127	Secretory carrier-associated membrane protein 2	SCAMP2	↓
O75398	Deformed epidermal autoregulatory factor 1 homolog	DEAF1	↓
Q13595	Transformer-2 protein homolog alpha	TRA2A	↑
O75915	PRA1 family protein 3	ARL6IP5	↑

Uniprot code	Protein names	Gene names (primary)	Expression
Q92564	DCN1-like protein 4	DCUN1D4	↑
Q7L5Y9	E3 ubiquitin-protein transferase MAEA	MAEA	↓
Q8WTX9	Palmitoyltransferase ZDHHC1	ZDHHC1	↓
Q9H492	Microtubule-associated proteins 1A/1B light chain 3A	MAP1LC3A	↓
O95622	Adenylate cyclase type 5	ADCY5	↓
P31483	Nucleolysin TIA-1 isoform p40	TIA1	↑
O00391	Sulfhydryl oxidase 1	QSOX1	↓
Q8N1F8	Serine/threonine-protein kinase 11-interacting protein	STK11IP	↓
Q9Y5U9	Immediate early response 3-interacting protein 1	IER3IP1	↑
Q04900	Sialomucin core protein 24	CD164	↑
Q9Y3C5	RING finger protein 11	RNF11	↑
P62820	Ras-related protein Rab-1A	RAB1A	↑
Q8N114	Protein shisa-5	SHISA5	↓
Q13424	Alpha-1-syntrophin	SNTA1	↓
Q9H6Y5	PDZ domain-containing protein MAGIX	MAGIX	↓
Q9HBH9	MAP kinase-interacting serine/threonine-protein kinase 2	MKNK2	↓
P51884	Lumican	LUM	↑
P61026	Ras-related protein Rab-10	RAB10	↑
O14521	Succinate dehydrogenase [ubiquinone] cytochrome b small subunit, mitochondrial	SDHD	↑
Q12982	BCL2/adenovirus E1B 19 kDa protein-interacting protein 2	BNIP2	↑
O00186	Syntaxin-binding protein 3	STXBP3	↑
Q86UZ6	Zinc finger and BTB domain-containing protein 46	ZBTB46	↓
Q9Y241	HIG1 domain family member 1A, mitochondrial	HIGD1A	↑
Q9Y592	Centrosomal protein of 83 kDa	CEP83	↑
P56589	Peroxisomal biogenesis factor 3	PEX3	↑
Q5BKX6	Solute carrier family 45 member 4	SLC45A4	↓
Q9H4M3	F-box only protein 44	FBXO44	↓
Q6PID6	Tetratricopeptide repeat protein 33	TTC33	↑
Q9UM82	Spermatogenesis-associated protein 2	SPATA2	↓
P51805	Plexin-A3	PLXNA3	↓
Q86WX3	Active regulator of SIRT1	RPS19BP1	↓
Q969V6	Myocardin-related transcription factor A	MRTFA	↓
Q8TDQ7	Glucosamine-6-phosphate isomerase 2	GNPDA2	↑
Q8IWT6	Volume-regulated anion channel subunit LRRC8A	LRRC8A	↓
P30533	Alpha-2-macroglobulin receptor-associated protein	LRPAP1	↓
P11279	Lysosome-associated membrane glycoprotein 1	LAMP1	↓
Q9NPC7	Myoneurin	MYNN	↑
P0C7P0	CDGSH iron-sulfur domain-containing protein 3, mitochondrial	CISD3	↓
P34059	N-acetylgalactosamine-6-sulfatase	GALNS	↓
O60568	Multifunctional procollagen lysine hydroxylase and glycosyltransferase LH3	PLOD3	↓

Uniprot code	Protein names	Gene names (primary)	Expression
Q07002	Cyclin-dependent kinase 18	CDK18	↓
O43759	Synaptogyrin-1	SYNGR1	↓
Q13554	Calcium/calmodulin-dependent protein kinase type II subunit beta	CAMK2B	↓
Q17RB0	Retrotransposon Gag-like protein 8B	RTL8B	↓
Q9BXS5	AP-1 complex subunit mu-1	AP1M1	↓
Q9NVE7	4'-phosphopantetheine phosphatase	PANK4	↓
P14678	Small nuclear ribonucleoprotein-associated proteins B and B'	SNRPB	↓
Q9NPJ6	Mediator of RNA polymerase II transcription subunit 4	MED4	↑
Q9GZN1	Actin-related protein 6	ACTR6	↑
O75676	Ribosomal protein S6 kinase alpha-4	RPS6KA4	↓
P31751	RAC-beta serine/threonine-protein kinase	AKT2	↓
Q8IZL8	Proline-, glutamic acid- and leucine-rich protein 1	PELP1	↓
O14544	Suppressor of cytokine signaling 6	SOCS6	↑
Q16718	NADH dehydrogenase [ubiquinone] 1 alpha subcomplex subunit 5	NDUFA5	↑
P21127	Cyclin-dependent kinase 11B	CDK11B	↓
P04075	Fructose-bisphosphate aldolase A	ALDOA	↓
Q9BTX1	Nucleoporin NDC1	NDC1	↑
Q99758	ATP-binding cassette sub-family A member 3	ABCA3	↓
Q9H9A6	Leucine-rich repeat-containing protein 40	LRRC40	↑
P56377	AP-1 complex subunit sigma-2	AP1S2	↑
Q9NZL9	Methionine adenosyltransferase 2 subunit beta	MAT2B	↑
O75154	Rab11 family-interacting protein 3	RAB11FIP3	↓
Q8NAT1	Protein O-linked-mannose beta-1,4-N-acetylglucosaminyltransferase 2	POMGNT2	↓
Q9UIV1	CCR4-NOT transcription complex subunit 7	CNOT7	↑
P17096	High mobility group protein HMG-I/HMG-Y	HMGA1	↓
O00258	Guided entry of tail-anchored proteins factor 1	GET1	↑
Q86X67	Nucleoside diphosphate-linked moiety X motif 13	NUDT13	↑
Q8WU17	E3 ubiquitin-protein ligase RNF139	RNF139	↑
A6NDG6	Glycerol-3-phosphate phosphatase	PGP	↓
O60493	Sorting nexin-3	SNX3	↑
P09001	39S ribosomal protein L3, mitochondrial	MRPL3	↑
Q7LGA3	Heparan sulfate 2-O-sulfotransferase 1	HS2ST1	↑
Q15170	Transcription elongation factor A protein-like 1	TCEAL1	↑
Q9BYV2	Tripartite motif-containing protein 54	TRIM54	↓
Q6NUQ1	RAD50-interacting protein 1	RINT1	↑
Q9NRR3	CDC42 small effector protein 2	CDC42SE2	↑
Q9GZU7	Carboxy-terminal domain RNA polymerase II polypeptide A small phosphatase 1	CTDSP1	↓
O95406	Protein cornichon homolog 1	CNIH1	↑
Q13724	Mannosyl-oligosaccharide glucosidase	MOGS	↓

Uniprot code	Protein names	Gene names (primary)	Expression
P18074	General transcription and DNA repair factor IIH helicase subunit XPD	ERCC2	↓
P53999	Activated RNA polymerase II transcriptional coactivator p15	SUB1	↑
Q5VSY0	G kinase-anchoring protein 1	GKAP1	↑
Q92797	Symplekin	SYMPK	↓
Q2TAY7	WD40 repeat-containing protein SMU1	SMU1	↑
Q9Y6A1	Protein O-mannosyl-transferase 1	POMT1	↓
O15269	Serine palmitoyltransferase 1	SPTLC1	↑
Q8TCF1	AN1-type zinc finger protein 1	ZFAND1	↑
Q9Y3C4	EKC/KEOPS complex subunit TPRKB	TPRKB	↑
Q9UNL2	Translocon-associated protein subunit gamma	SSR3	↑
Q8IWZ8	SURP and G-patch domain-containing protein 1	SUGP1	↓
Q7L2H7	Eukaryotic translation initiation factor 3 subunit M	EIF3M	↑
Q9UJV9	Probable ATP-dependent RNA helicase DDX41	DDX41	↓
O15357	Phosphatidylinositol 3,4,5-trisphosphate 5-phosphatase 2	INPPL1	↓
Q9HA65	TBC1 domain family member 17	TBC1D17	↓
O94777	Dolichol phosphate-mannose biosynthesis regulatory protein	DPM2	↓
Q9NW81	Distal membrane-arm assembly complex protein 2	DMAC2	↓
P23193	Transcription elongation factor A protein 1	TCEA1	↑
Q15185	Prostaglandin E synthase 3	PTGES3	↑
O43390	Heterogeneous nuclear ribonucleoprotein R	HNRNPR	↑
Q8TB24	Ras and Rab interactor 3	RIN3	↓
Q3MII6	TBC1 domain family member 25	TBC1D25	↓
Q9UK59	Lariat debranching enzyme	DBR1	↑
P51688	N-sulphoglucosamine sulphohydrolase	SGSH	↓
P50336	Protoporphyrinogen oxidase	PPOX	↓
Q96GX9	Methylthioribulose-1-phosphate dehydratase	APIP	↑
P14868	Aspartate--tRNA ligase, cytoplasmic	DARS1	↑
P40189	Interleukin-6 receptor subunit beta	IL6ST	↑
Q9HD45	Transmembrane 9 superfamily member 3	TM9SF3	↑
O00506	Serine/threonine-protein kinase 25	STK25	↓
P26640	Valine--tRNA ligase	VARS1	↓
Q8TBB0	THAP domain-containing protein 6	THAP6	↑
Q9BYG3	MKI67 FHA domain-interacting nucleolar phosphoprotein	NIFK	↑
O75159	Suppressor of cytokine signaling 5	SOCS5	↑
O60613	Selenoprotein F	SELENOF	↑
P43694	Transcription factor GATA-4	GATA4	↓
Q499Z4	Zinc finger protein 672	ZNF672	↓
Q8N2K1	Ubiquitin-conjugating enzyme E2 J2	UBE2J2	↓
Q5VW32	BRO1 domain-containing protein BROX	BROX	↑
Q86X55	Histone-arginine methyltransferase CARM1	CARM1	↓

Uniprot code	Protein names	Gene names (primary)	Expression
Q5JTJ3	Cytochrome c oxidase assembly factor 6 homolog	COA6	↑
Q8WVJ2	NudC domain-containing protein 2	NUDCD2	↑
Q9BUE6	Iron-sulfur cluster assembly 1 homolog, mitochondrial	ISCA1	↑
P84077	ADP-ribosylation factor 1	ARF1	↓
Q96EB6	NAD-dependent protein deacetylase sirtuin-1	SIRT1	↑
Q9BY44	Eukaryotic translation initiation factor 2A	EIF2A	↑
P26572	Alpha-1,3-mannosyl-glycoprotein 2-beta-N-acetylglucosaminyltransferase	MGAT1	↓
Q9Y3T6	R3H and coiled-coil domain-containing protein 1	R3HCC1	↓
O94782	Ubiquitin carboxyl-terminal hydrolase 1	USP1	↑
Q9P055	JNK1/MAPK8-associated membrane protein	JKAMP	↑
P61088	Ubiquitin-conjugating enzyme E2 N	UBE2N	↑
Q00536	Cyclin-dependent kinase 16	CDK16	↓
Q96IY1	Kinetochore-associated protein NSL1 homolog	NSL1	↑
Q13185	Chromobox protein homolog 3	CBX3	↑
Q86WP2	Vasculin	GPBP1	↑
Q9H1Y0	Autophagy protein 5	ATG5	↑
Q96Q45	Transmembrane protein 237	TMEM237	↑
O95573	Long-chain-fatty-acid--CoA ligase 3	ACSL3	↑
O15243	Leptin receptor gene-related protein	LEPROT	↑
Q9UHY1	Nuclear receptor-binding protein	NRBP1	↓
P50579	Methionine aminopeptidase 2	METAP2	↑

Supplementary Table S3: Profile of drugs studied in HFpEF according to DrugBank database.

Drug	Target Protein Name	Target Gene Name
**Enalapril**	Angiotensin-converting enzyme	ACE
**Captopril**	Angiotensin-converting enzyme	ACE
**Ramipril**	Angiotensin-converting enzyme	ACE
**Losartan**	Angiotensin II type-1 receptor, AT1	AGTR1
**Valsartan**	Angiotensin II type-1 receptor, AT1	AGTR1
**Sacubitril/Valsartan**	Angiotensin-receptor neprilysin inhibitor	NEP/AGTR1
**Bisoprolol**	Beta-1 adrenergic receptor	ADRB1
**Spironolactone**	Mineralocorticoid receptor, MR	NR3C2
**Eplerenone**	Mineralocorticoid receptor, MR	NR3C2

<u>Supplementary Table S4</u>: Empagliflozin drug targets considered. Red arrow (↓) indicates an inhibition by the drug.

UniProt ID	Protein Name	Gene Name	Effect
P31639	**Sodium/glucose cotransporter 2**	SLC5A2 (SGLT2)	↓
P19634	**Sodium/hydrogen exchanger 1**	SLC9A1 (NHE1)	↓
P48764	**Sodium/hydrogen exchanger 3**	SLC9A3 (NHE3)	↓

Supplementary Table S5: Empagliflozin bioflags considered. The "effect" column indicates whether the drug induces an inhibition (↓) or an activation (↑) on said bioflag.

Uniprot	Protein Name	Gene Name	Effect
P40763	**Signal transducer and activator of transcription 3**	STAT3	↑
P35228	**Nitric oxide synthase, inducible**	NOS2	↓
P05231	**Interleukin-6**	IL6	↓
Q02338	**D-beta-hydroxybutyrate dehydrogenase, mitochondrial**	BDH1	↑
P01579	**Interferon gamma**	IFNG	↓
P05091	**Aldehyde dehydrogenase, mitochondrial**	ALDH2	↑
P01275	**Glucagon**	GCG	↑
P01308	**Insulin**	INS	↓
Q9BYF1	**Angiotensin-converting enzyme 2**	ACE2	↑
P23560	**Brain-derived neurotrophic factor**	BDNF	↑
Q13547	**Histone deacetylase 1**	HDAC1	↓
Q92769	**Histone deacetylase 2**	HDAC2	↓
O15379	**Histone deacetylase 3**	HDAC3	↓
Q9BY41	**Histone deacetylase 8**	HDAC8	↓
P01584	**Interleukin-1 beta**	IL1B	↓
Q96P20	**NACHT, LRR and PYD domains-containing protein 3**	NLRP3	↓
P29466	**Caspase-1**	CASP1	↓
P01375	**Tumor necrosis factor**	TNF	↓
Q9H3M7	**Thioredoxin-interacting protein**	TXNIP	↓
Q9UBK2	**Peroxisome proliferator-activated receptor gamma coactivator 1-alpha**	PRGC1	↑
Q8WZ42	**Titin**	TTN	↑
P19429	**Troponin I, cardiac muscle**	TNNI3	↑
Q14896	**Myosin-binding protein C, cardiac-type**	MYBPC3	↑
P01137	**Transforming growth factor beta-1 proprotein**	TGFB1	↓
P02452	**Collagen alpha-1(I) chain**	COL1A1	↓
P08123	**Collagen alpha-2(I) chain**	COL1A2	↓
P68133	**Actin, alpha skeletal muscle**	ACTA1	↓
P29279	**Connective tissue growth factor**	CTGF	↓
P08253	**Matrix metalloproteinase 2**	MMP2	↓

Supplementary Table S6. Demographic, clinical characteristics of included patients.

HFpEF cohort	N=15
Age, years	69 ± 12
Male	12 (80)
White	15 (100)
HF duration, years	6.3 ± 3.4
NYHA class	
I	2 (13.3)
II	13 (86.7)
LVEF, %	58 ± 6
Diabetes	15 (100)
Hypertension	10 (66.7)
COPD	1 (6.7)
Smoker or past smoker	8 (53.3)
BMI, Kg/m2	32.3 ± 3.5
Treatment	
ACEI/ARB	11 (73.3)
Betablockers	14 (93.3)
MRA	12 (80.0)
Loop diuretics	10 (66.7)

Supplementary Figure S1: Input-output signals within the mathematical models.

The models resemble a Multilayer Perceptron of an Artificial Neural Network over the human protein network (where neurons are the proteins, and the edges of the network are used to transfer the information). This methodology is used for describing all plausible relationships between an input (or *stimulus*, in this case empagliflozin's target proteins) and an output (or *response*, in this case HFpEF protein effectors).

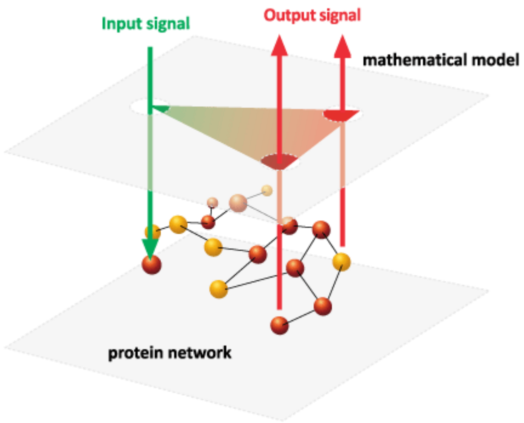

Supplementary Figure S2. Graphical representation of the mechanism of action.

Genes and proteins appear as white, circled nodes. Interactions are represented as green arrows for activation interactions, red lines for inhibition interactions and blue diamond-ended lines for unspecified interactions (in case they are represented).

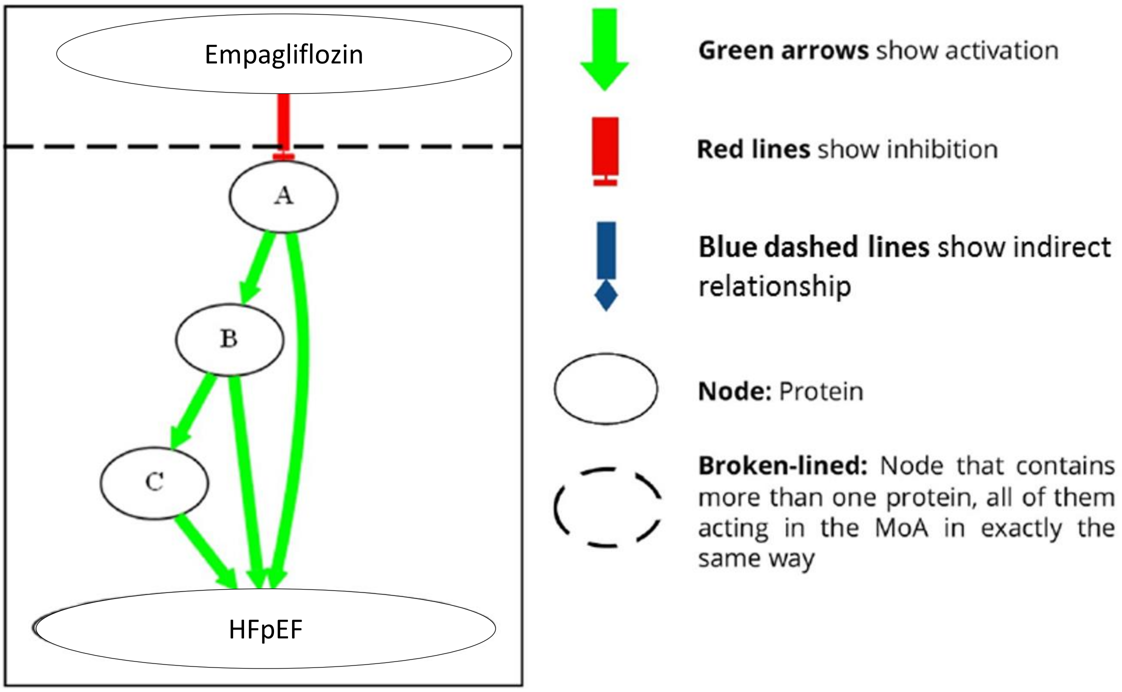

Supplement: Supplementary file 1 — Supplementary Information. [file 41598_2021_91546_MOESM1_ESM.pdf]
